# Optimising the short and long-term clinical outcomes for koalas (*Phascolarctos cinereus*) during treatment for chlamydial infection and disease

**DOI:** 10.1371/journal.pone.0209679

**Published:** 2018-12-27

**Authors:** Amy Robbins, Joanne Loader, Peter Timms, Jonathan Hanger

**Affiliations:** 1 Endeavour Veterinary Ecology Pty Ltd, Toorbul, Queensland, Australia; 2 Genecology Centre, Faculty of Science, Health, Education and Engineering, University of the Sunshine Coast, Sippy Downs, Queensland, Australia; Universita degli Studi di Bologna, ITALY

## Abstract

Koalas (*Phascolarctos cinereus*) have suffered severe declines in the northern extent of their range due to a variety of threats, including habitat destruction, trauma from cars and dogs, climate change and importantly, disease. The most significant pathogen in koalas is *Chlamydia pecorum*, which causes inflammation and fibrosis at mucosal sites, resulting in blindness, infertility and death in severe cases. *Chlamydia* treatment can be problematic in koalas as the response to treatment is often poor in chronic cases and antimicrobial choice is limited. Thus, chlamydial disease is a severely threatening process for koala conservation. We investigated the short and long-term clinical outcomes for 167 koalas with *Chlamydia* that underwent capture, telemetric monitoring and intensive veterinary management as part of a large-scale population management program in South East Queensland. *Chlamydia* treatments included the standard regimen of daily subcutaneous chloramphenicol injections (60mg/kg) for 14 to 28-days, and a variety of non-standard regimens such as topical antimicrobials only (for ocular disease), surgical treatment only (for bilateral reproductive tract disease), and other antimicrobials/treatment lengths. To assess these regimens we analysed clinical records, field monitoring data and swab samples collected from the urogenital tract and ocular conjunctiva. Overall, in contrast to other studies, treatment was generally successful with 86.3% of treated koalas released back into the wild. The success of treatment rose to 94.8% however, when the standard treatment regimen was employed. Further, 100% of koalas that were also treated with surgical ovariohysterectomy (n = 12) remained healthy for a median of 466 days of post-treatment monitoring, demonstrating the benefits of surgical treatment. Previous studies reported 45-day chloramphenicol regimens, but the shorter standard regimen still achieved microbiological cure and reduces the risk of negative sequelae associated with treatment and/or captivity and treatment costs. Despite these positive clinical outcomes, alternatives to chloramphenicol are warranted due to its decreasing availability.

## Introduction

Chlamydial infections caused by *Chlamydia pecorum*, and less commonly *C*. *pneumoniae*, are endemic in almost all free-living koala populations, with variable impacts on koala health [1–4]. In some populations, particularly in Queensland (Qld) and New South Wales (NSW) where koalas are suffering severe declines, chlamydial disease has significant negative impacts on koala welfare, fecundity and survival [5–7]. In South East Qld (SE Qld) for example, the prevalence of infection and disease varies between sub-populations but can be as high as 85% and 52.5% respectively, with some studies reporting infertility in up to 57% of the females examined [1, 7, 8]. Because of these impacts on morbidity, mortality and fecundity, chlamydial disease can substantially contribute to population declines and localised extinctions and is a severely threatening process for the conservation of the koala [9, 10].

The classical manifestations of chlamydial disease in koalas are ocular disease, urinary tract disease, reproductive tract disease, and less commonly, respiratory tract disease (see [2] for a review of chlamydial disease in koalas). Acute chlamydial disease is characterised by a mainly neutrophilic cellular response, whereas a prolific mononuclear inflammatory cell response dominated by plasma cells, in addition to mucosal hyperplasia and fibrosis, characterises chronic disease [11, 12]. Consequently, chronic disease can result in significant changes to normal anatomy and loss of function causing temporary or permanent blindness (in severe cases of keratoconjunctivitis), and incontinence and sterility (due to urogenital tract disease). Koalas in the acute phase of disease therefore show subtler clinical signs and are less likely to be detected and captured for treatment. Hence, most free-living koalas presented to care facilities for chlamydial illness are suffering from chronic disease, complicating treatment.

The aim of treatment of chlamydial disease is to achieve a microbiological cure and reduce inflammation and fibrosis to a level at which discomfort and structural changes are minimal so that return of function is sufficient for good prospects of survival in the wild. Antimicrobials are considered to be the first line of treatment, but anti-inflammatories, surgical intervention and vaccination all offer therapeutic benefits [13]. While several antibiotics, such as the tetracyclines and macrolides, have shown efficacy in the treatment of *Chlamydia* in other species, they have not been routinely used in koalas due to the risk of causing fatal changes to the koala’s specialised gastrointestinal microflora [14, 15]. Florfenicol, an analogue of chloramphenicol, is reported to have good activity against *Chlamydia in vitro* [16] prompting investigation as an alternative in a recent study by Budd *et al*. [17]. However, only suboptimal plasma concentrations were achieved after administration of doses tolerated by koalas (20mg/kg SC), resulting in equivocal clinical outcomes and an increased risk of gastrointestinal dysbiosis. Hence, treatment options are limited.

Only two antibiotics are commonly used to treat *Chlamydia* in koalas: chloramphenicol and enrofloxacin. Recent research indicates that enrofloxacin, whilst generally considered safe for use in koalas and anecdotally reported to be efficacious, is not able to reach effective plasma concentrations after subcutaneous or oral administration at doses avoiding toxicity and is not able to achieve microbiological cure [18–20]. Hence, by exclusion, chloramphenicol is the mainstay of the currently accepted treatment regimen for *Chlamydia* in koalas. When administered daily by subcutaneous injection at a dose rate of 60mg/kg, it results in marked and rapid reduction in the shedding of *Chlamydia* from ocular and urogenital sites, with no relapse for up to 10 weeks post treatment [21, 22]. In addition, Black *et al*. [20] reported that chloramphenicol was efficacious against *Chlamydia* in koalas based on its pharmacokinetic profile and *in vitro* susceptibility.

Despite the evidence outlined above for the efficacy of chloramphenicol at achieving a microbiological cure for *Chlamydia* in koalas, optimal clinical outcomes are not always reported with this treatment. Due to the degree of inflammation, hyperplasia and fibrosis resulting from chronic infection with *Chlamydia*, and suspected on-going immunopathology, antimicrobial treatment alone may be ineffective at resolving clinical signs [22]. Furthermore, equivocal clinical outcomes and negative sequelae have been reported with chloramphenicol use in koalas [23, 24]. In a study by Govendir *et al*. [22] for example, only two of five animals with urogenital chlamydiosis survived beyond the duration of chloramphenicol treatment. Further, there has been little longitudinal monitoring of koalas after treatment for chlamydial infection and disease, or investigation of the long-term effects of the surgical treatment, ovariohysterectomy, for reproductive disease.

Hence, we sought to determine the effectiveness of the treatment regimens employed during a large-scale koala management program in SE Qld, and the conservation benefits of intensive population-scale veterinary management in a declining koala population. We report here, the short and long-term clinical outcomes for 167 free-living koalas presenting with chlamydial infection and disease.

## Materials and methods

### Koalas and study site

A koala management program was conducted between 2013 and 2017 by the Qld Department of Transport and Main Roads as part of the Moreton Bay Rail (MBR) construction project in SE Qld. The aim of the program was to mitigate and manage potential impacts of the construction and operation of the new 13 km rail project between Petrie and Kippa-Ring on resident koalas inhabiting blocks of remnant koala habitat bisected and adjacent to the rail line. The program involved the capture, telemetric monitoring and veterinary management of 503 free-living independent and near-independent koalas (217 males, 285 females and 1 intersex), an estimated 95% of the resident population [7].

### Monitoring of koalas

Koalas weighing more than 3 kg were monitored using near-real-time biotelemetry devices (*K-Tracker* telemetry system, LX Group, Sydney, New South Wales (NSW)). This telemetry system reported global positioning system (GPS) locations and activity data from each of the tagged koalas via 12-hourly data uploads to an internet-based server. Koalas wearing *K-Tracker* biotelemetry collars were therefore able to be monitored remotely via the internet but were also field-tracked using very high frequency (VHF) radio telemetry at least once per fortnight. Koalas between 1 kg and 3 kg were not large enough to be fitted with the *K-Tracker* collars, so were field-tracked using VHF radio telemetry several times a week. Koalas were monitored more frequently if there were health or welfare concerns, or if activity data reported by *K-Tracker* collars indicated low or zero activity. At each field monitoring event, koalas were examined with binoculars and various data recorded, including comments on external signs of health and the presence/absence of joeys. Koalas displaying clinical signs of illness were recaptured as soon as practicable for veterinary treatment.

### Husbandry

Koalas admitted for treatment were housed individually in purpose-designed enclosures of approximately 3 m x 2.5 m x 2.5 m (height) at the Endeavour Veterinary Ecology (EVE) facilities in Toorbul, Queensland (with some early cases treated at the Australia Zoo Wildlife Hospital (AZWH) in Beerwah, Queensland). Wall-mounted brackets supported two timber perches connected by a horizontal branch. Fresh browse of koala-edible species including *Eucalyptus spp*., *Corymbia spp*., *Melaleuca spp*., and *Lophostemon spp*. were provided daily, along with *ad lib* water and termite mound. Scat number and quality were recorded daily, in addition to scores for browse eaten, body condition, hydration, demeanour and signs of pain.

### Clinical examinations and sampling

Thorough and standardised clinical examinations were conducted on all koalas after initial capture, at 6-monthly intervals (or sooner if clinical signs of ill-health were detected during field monitoring), and at the cessation of monitoring/conclusion of the program [7]. Clinical examinations were conducted with alfaxalone (Alfaxan, Jurox, Rutherford, NSW) sedation (dose rate 3-5mg/kg IM), occasionally supplemented with oxygen and isoflurane (Isoflo, Abbott, Botany, NSW) if required. All examinations included a thorough physical examination, sonographic examination of the urogenital tract (including kidneys), and cytological examination of blood, bone marrow, peritoneal fluid, and urine sediment. Additional diagnostic tests, such as radiography and routine biochemistry, were occasionally performed if indicated by the findings of the clinical examination.

At all clinical examinations a Clearview *Chlamydia* Antigen test (Alere, Melbourne, Victoria) was carried out, according to the manufacturer’s instructions, on swab samples collected from the ocular conjunctiva, urogenital sinus (females) or urethra (males), and urine sediment (when possible) with rayon-tipped aluminium-shafted swabs (Copan, Murrieta, California). This test was withdrawn from the market in late 2016 and was not available for the final 142 examinations. Swab samples from the ocular conjunctiva and urogenital tract were also collected for analysis by real-time polymerase chain reaction (qPCR) but results were not available sufficiently promptly to influence clinical management of cases (except for one). Hence, although the Clearview *Chlamydia* Antigen test (Alere, Melbourne, Victoria) has been criticised for poor sensitivity [23], it was considered a valuable point-of-care test to identify infection/shedding of *Chlamydia* in apparently healthy koalas [25]. Most veterinary procedures were conducted at the EVE facilities in Toorbul, Queensland, by koala-experienced veterinary staff to ensure consistency. Data were recorded in a purpose-designed database, using the FileMaker software (Apple, Sydney, NSW).

### PCR diagnosis of *Chlamydia*

Swab samples collected from the ocular conjunctiva and urogenital tract during clinical examinations were stored at -20°C until processing. DNA was extracted using a QIAamp DNA mini kit (Qiagen, Chadstone, Victoria) according to the manufacturer’s instructions. The extracted samples were analysed using qPCR methods modified from Jelocnik *et al*. [26] targeting a 209 base pair region of a *C*. *pecorum*-specific conserved gene CpecG_0573. Specifically, the qPCR assays were carried out in a total volume of 10μL, consisting of 5μL iTaq Universal SYBR Green Supermix (Bio-Rad, Gladesville, New South Wales), 1.5μL DNA/RNA-free water, 0.5μL of each 10μM forward and reverse primer, and 2.5μL DNA template. Samples were run in duplicate, and positive and negative controls (DNA/RNA free water and miliqH2O) were included in all qPCR assays (no inhibition controls–previously tested, well-established protocol). Samples with less than 50 copies/μL or amplifying at more than 32 cycles were considered to be below the detectable limit of the assay.

### Treatment of chlamydial infections and disease

Koalas with chlamydial disease and/or a positive Clearview test result (≥ 2+ positive–see [7]) were admitted for treatment or euthanized if the prognosis was poor. Suitability for treatment was determined with regard to age, severity of disease (including suspected sterility), presence of pouch young, and long-term prognosis. Koalas requiring euthanasia on welfare grounds were administered an intravenous injection of barbiturate solution (Lethabarb, Virbac, Milperra, NSW), prior to recovery from anaesthesia. Koalas admitted for treatment received a 14 to 28-day course of daily subcutaneous chloramphenicol injections (Chloramphenicol 150, Ceva, Glenorie, NSW), administered at a dose rate of 60mg/kg (the standard regimen). Some koalas were released prior to completing a 28-day course of chloramphenicol injections due to welfare concerns, because of limited availability of chloramphenicol, when mild to moderate ocular disease had resolved, or they were being treated for Clearview test positivity only (with no clinically detectable disease). A minimum treatment course of 14 days was deemed necessary to achieve a microbiological cure (qPCR negativity), based on previous studies indicating that the shedding of *Chlamydia* ceased (or was significantly reduced) after this length of treatment [21, 22].

Additional treatment for koalas with keratoconjunctivitis (and no evidence of corneal ulceration) included chloramphenicol/hydrocortisone (Chloroptsone, Ceva, Glenorie, NSW) eye ointment topically twice daily in the affected eye(s) until there was a substantial improvement in clinical signs (usually one to two weeks), and dexamethasone (Maxidex, Alcon, Frenchs Forest, NSW) eye drops topically twice daily until there was substantial resolution of the proliferative tissue response/inflammation in the conjunctiva (usually approximately one week). Most koalas would not tolerate ocular medication for more than one to two weeks, and if an animal became distressed by topical therapy it was discontinued. If there was evidence of pain during urination (as evidenced by vocalisation during urination), koalas were administered paracetamol (Panadol Children, GlaxoSmithKline, Abbotsford, Victoria) at a dose rate of 15mg/kg per os, two or three times daily until signs of pain resolved. Koalas with particularly severe inflammatory changes in the bladder (based on sonographic findings), or severe pain during urination (as evidenced by marked, frequent vocalisation during urination), were treated with oral prednisolone (Redipred, Aspen, St Leonards, NSW) at a dose rate of 0.5-1mg/kg per os, once or twice daily for one to two weeks, with a staged reduction over a period of one to two weeks.

Supportive care, such as supplementary feeding, oral water administration and bathing of the rump and eyes to remove exudate and debris, was also provided. Koalas were monitored closely for candidiasis by regular microscopic examination of faecal wet mounts and were treated with 1 tablet of amphotericin B (Fungilin Lozenges, Bristol-Myers Squibb, Mulgrave, Victoria) mixed with water per os, four times daily when necessary. In addition, if koalas developed oxalate nephrosis whilst hospitalised a treatment protocol was adopted in the latter stages of the program that included oral transfaunation with fresh caecal contents (minimum of 4mL per day for 2 to 5 days), and oral (up to 50mL three times daily) and parenteral fluid administration (mostly administered subcutaneously, every other day). Finally, koalas were generally examined under anaesthesia at least once during the treatment period (often once per week), and at the completion of treatment prior to their release back into the wild.

The veterinary preparation of injectable chloramphenicol was temporarily unavailable in 2013/2014 and during this time some koalas were admitted for non-standard treatments. These included topical antimicrobial treatment only in cases of keratoconjunctivitis, surgical ovariohysterectomy only in cases of bilateral reproductive disease, treatment with other antimicrobials (e.g. florfenicol (Nuflor, Schering-Plough Animal Health Limited, Bendigo, Victoria–reported in [17]) or short chloramphenicol treatment lengths (<14 days). In addition, due to the lack of an effective antimicrobial treatment for *Chlamydia* during this time, some koalas that may have otherwise been admitted for treatment had chloramphenicol been available were either released back into the wild despite the detection of *Chlamydia* infection or disease (and then recaptured for treatment once chloramphenicol was available) or were euthanized on welfare grounds in more severe or complicated cases.

### Statistical analyses

Differences between the sexes with regard to clinical outcomes and the detection and prevalence of chlamydial disease were analysed with a two-tailed Fisher’s exact test, as was the efficacy of different lengths of chloramphenicol treatment (within the standard regimen), using the GraphPad software. Differences in the median inter-admission intervals between the sexes were analysed using a Mann-Whitney U test. Statistical significance of all tests was concluded at *p-values* ≤ 0.05.

### Regulatory approvals

The koala management program was conducted under approvals issued by the Queensland Department of Agriculture and Fisheries (approvals CA 2012/03/597, CA 2013/09/719, CA 2014/06/777, CA 2015/03/852, and CA 2016/03/950). Work with koalas was authorised by scientific purposes permits issued by the Queensland Department of Environment and Heritage Protection (approvals WISP 11525212, WISP 16125415, WISP 13661313, WITK 14173714 and WISP 17273716). Swab samples were analysed under approval number AN/A/13/80 issued by the University of the Sunshine Coast Animal Ethics Committee.

## Results

### Overview of the koala management program

In total, 503 free-living koalas were included in the koala management program over the four-year period between 2013 and 2017, and 56.7% were female (n = 285), 43.1% were male (n = 217), and 1 koala was intersex. Of these, 167 (33.2%) were either diagnosed with chlamydial disease or were *Chlamydia* antigen-positive. Chlamydial disease was diagnosed in 158 koalas and 9 koalas were antigen-positive but clinically normal (4 females and 5 males). Overall, chlamydial disease was not significantly more common in either sex (*p* = 0.1208) and was detected in 98 females (34.4% of females) and 60 males (27.7% of males). The prevalence of disease at the first clinical examination was 21.1% in females (n = 60), and 14.8% in males (n = 32), and the remainder developed disease at some time after their first clinical examination during the period of their monitoring (n = 66) (Table 1).

**Table 1:** Summary of clinical outcomes for koalas with chlamydial disease or *Chlamydia* antigen positivity only during the koala management program.

Overview of outcomes (n = 503)	Female	Male
Koalas with chlamydial disease or Clearview positivity	102	65
Koalas euthanized at first exam	17	3
Koalas admitted for treatment	72	52
Koalas admitted more than once for treatment	28	14
Total number of admissions for treatment	100	66
Admissions released after treatment	88	61
Clearview positivity/qPCR load admissions	8	4
Chlamydial disease admissions	92	62
Median inter-admission interval (days)	205	300
Koalas euthanized during treatment	11	5
Koalas that died during treatment	1	0
Treated koalas that died/were euthanized shortly after release	3	3
Koalas with an unknown outcome after release	6	6

### Short and long-term outcomes of treatment

Of the 167 koalas in which chlamydial disease or *Chlamydia* antigen test positivity only was detected, 124 (74.2%, 72 females and 52 males) were suitable for admission for treatment. A further 28 koalas (16.8%, 22 females and 6 males) were euthanized at the time of the clinical examination at which disease was detected, and this occurred not quite significantly more often in females than males (*p* = 0.0545). The remaining 15 koalas (9%, 8 females and 7 males) were released without treatment because an effective treatment was not available (during the chloramphenicol shortage), and their clinical examination findings were not severe enough to warrant euthanasia on welfare grounds.

Most cases had positive short-term outcomes, with 86.3% of the koalas admitted for treatment being released back into the wild (n = 107). The likelihood of a positive short-term outcome was not significantly different between the sexes (*p* = 0.3009), with 90.4% of males (n = 47) and 83.3% of females (n = 60) released back into the wild after treatment. Of the cases that had negative short-term outcomes (n = 17), 12.9% (11 females, and 5 males) were euthanized during treatment and 1 female died during treatment (0.8%). An additional 4.8% (n = 6) of koalas died or were euthanized within one month of their release back into the wild because of illness that may have been a complication of their treatment (such as oxalate nephrosis).

Koalas were considered to have had positive outcomes after treatment when clinical signs of disease had resolved, completely, or sufficiently to justify release, and/or were *Chlamydia* antigen-negative. In some cases, koalas were released with mild residual signs of disease after receiving treatment, and this was most common for urogenital tract disease (e.g. mildly thickened bladder wall, or mild haematuria/inflammatory urine sediment). In the few cases where this occurred, and the koala had received an appropriate course of chloramphenicol (14 to 28 days), any residual clinical signs of disease had resolved at the subsequent clinical examination (usually one to six months later). Results from qPCR analysis of swab samples collected from the urogenital tract and ocular conjunctiva indicated that a 14-day course of chloramphenicol was sufficient to reduce shedding of *C*. *pecorum* to below detectable limits, resulting in a microbiological cure (S1 Table).

### Causes of negative short and long-term outcomes

**Fig 1:**
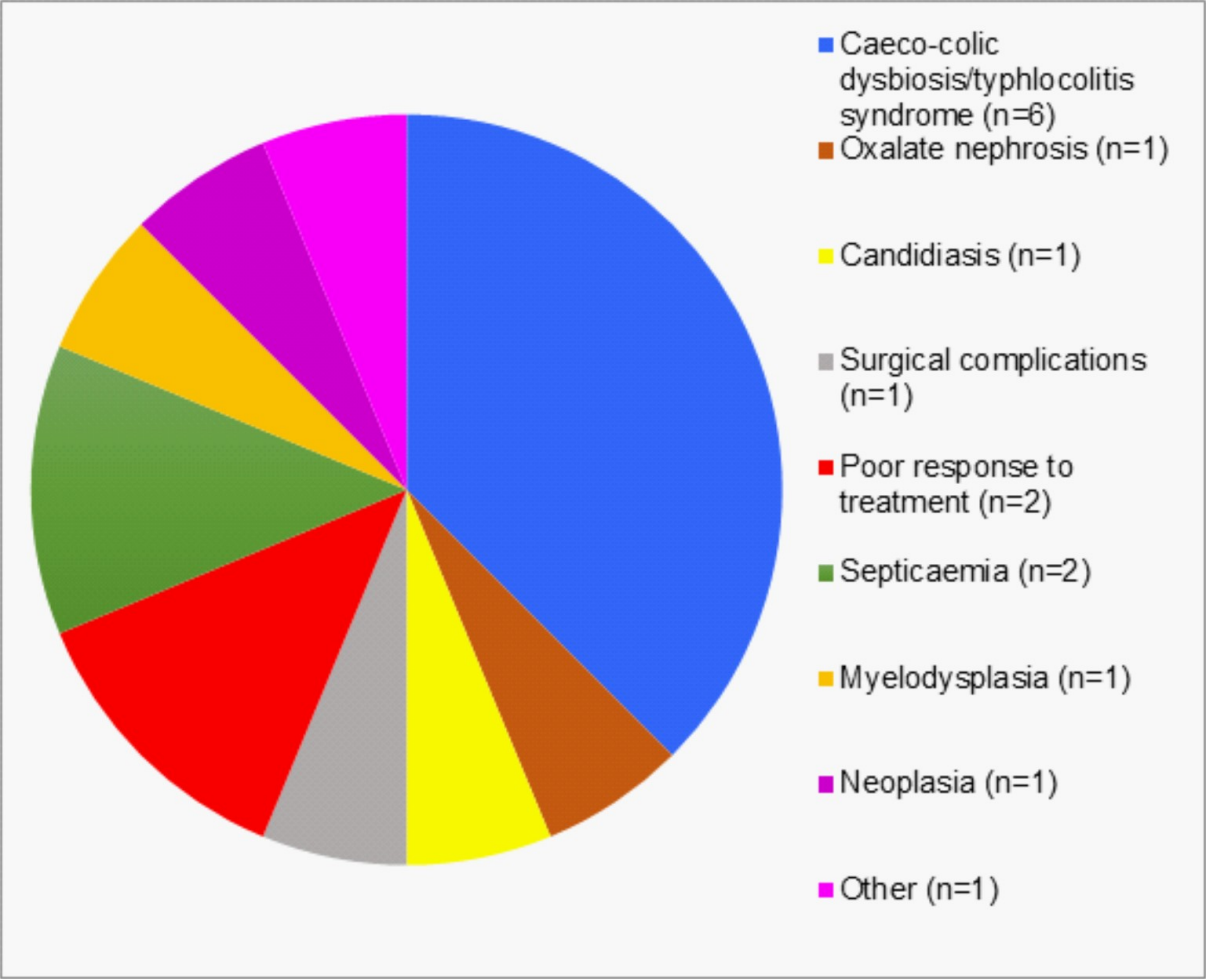
Reasons for negative short-term outcomes for koalas resulting in euthanasia during treatment of chlamydial disease or *Chlamydia* antigen test positivity only in the koala management program.

The main reason for negative short-term outcomes during treatment, resulting in euthanasia (n = 16), was the development of a concurrent illness warranting euthanasia (Fig 1). Concurrent illness resulted in the euthanasia of 87.5% of koalas with negative short-term outcomes (n = 14) and was not significantly more common in either sex (*p* = 0.1499), with 15.3% of females (n = 11) and 5.8% of males (n = 3) requiring euthanasia. The remaining 12.5% of koalas were euthanized due to a poor response to treatment (n = 2, males). Of those koalas that developed a concurrent illness warranting euthanasia, 64.3% were euthanized primarily due to iatrogenic conditions, considered to be negative sequelae of treatment and/or captivity (n = 9). These included caeco-colic dysbiosis/typhlocolitis syndrome (CDTS) (n = 6), which was diagnosed in 6.9% of females (n = 5) and 1.9% of males (n = 1), oxalate nephrosis (n = 1, female), candidiasis (n = 1, male), and complications following surgery (n = 1, female). An intestinal volvulus in 1 female koala was responsible for the only death during treatment. Of those koalas that had negative long-term outcomes in the month following their release, which may have been associated with their treatment (n = 6), 66.7% were due to the development of an iatrogenic condition (n = 4), namely CDTS (n = 2, 1 female and 1 male) and oxalate nephrosis (n = 2, 1 female and 1 male).

### Hospital admissions

During the koala management program, a total of 166 hospital admissions occurred for the treatment of chlamydial disease or *Chlamydia* antigen test positivity only. Multiple hospital admissions were required for 38.9% of females (n = 28) and 26.9% of males (n = 14), and this difference was not statistically significant (*p* = 0.1831). Of the koalas admitted multiple times for treatment, 37 koalas were admitted twice, 89.3% of females (n = 25) and 85.7% of males (n = 12); 4 koalas were admitted three times, 10.7% of females (n = 3) and 7.1% of males (n = 1); and 1 male koala was admitted four times for treatment (7.1% of males). There was a median number of 216 days between admissions for treatment (range 16–1001 days), and the median inter-admission interval was not significantly different between females and males (*p* = 0.41794) (205 days (range 16–1001 days) and 300 days (range 40–781 days), respectively).

### Reasons for multiple hospital admissions

**Fig 2:**
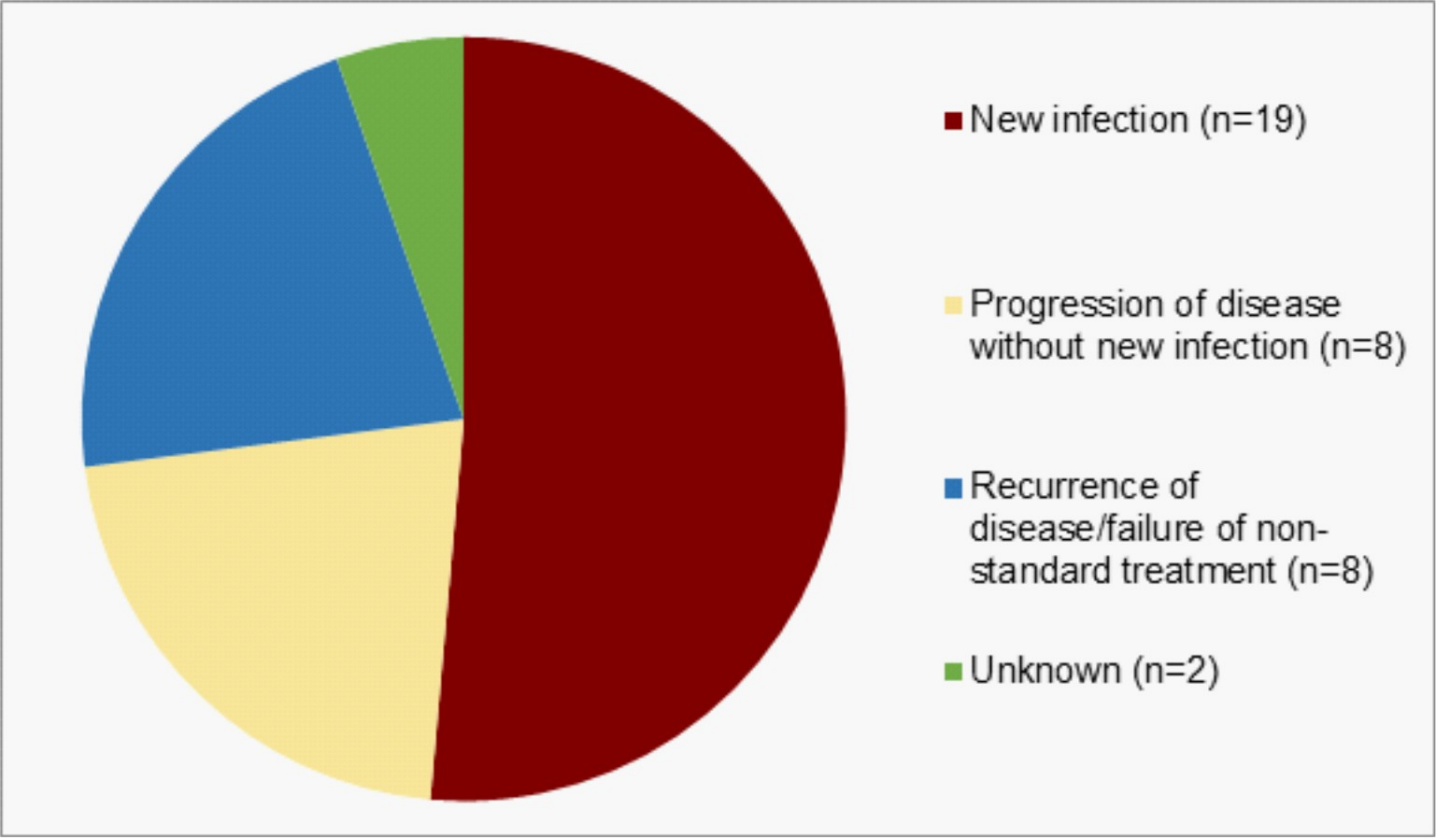
Reasons for multiple hospital admissions for the treatment of chlamydial disease or *Chlamydia* antigen test positivity only in the koala management program.

Clinical data (resolution of disease), inter-admission interval, qPCR results (negative at their post-treatment examination) and field data (reproductive status or history of contact with a diseased koala) were used to infer whether repeat hospital admissions were because of new infections, or recurrence/progression of a previously treated illness (S1 Table, S1 File). Of the 37 koalas treated on two separate occasions, 51.4% were likely due to new infections (n = 19, 12 females and 7 males), 21.6% were due to the progression of disease sequelae without new infections (e.g. development of bilateral reproductive cysts) (n = 8, all female), 21.6% (n = 8, 3 females and 5 males) were due to the recurrence of disease/failure of non-standard treatments (e.g. short treatment length, treatment with non-chloramphenicol antimicrobials, topical antimicrobial or surgical treatment only), and in 5.4% (2 cases, both female) of cases the reason for readmission was not able to be determined (Fig 2). Of the four koalas treated on three separate occasions, one female was treated three times consecutively, each time for a newly acquired infection, two females required additional treatment for disease progression without new or recrudescent infections and one male required two additional separate treatments due to failure of a non-standard (non-chloramphenicol) treatment. One male koala required four separate hospital admissions over a period of 4 years for the treatment of what we considered to be new infections on each occasion.

### Chloramphenicol treatment at Endeavour Veterinary Ecology

When the data were censored to remove non-standard treatments (e.g. short treatment length, treatment with non-chloramphenicol antimicrobials, topical antimicrobial or surgical treatment only), and those that took place at facilities other than EVE, the success of treatment of chlamydial disease rose. A total of 58 koalas, 45.8% of females (n = 33) and 48.1% of males (n = 25), were admitted for treatment with the standard course (14 to 28 days) of the veterinary preparation of injectable chloramphenicol at EVE. Of these, 94.8% were released back into the wild after treatment (n = 55, 90.9% of females (n = 30) and 100% of males (n = 25)), and 5.2% were euthanized during treatment (n = 3, 9.1% of females), 1.7% due to surgical complications (n = 1) and 3.5% due to the development of concurrent illness (n = 2).

In total, 7 of the koalas (12.1%) treated at EVE required multiple hospital admissions for treatment. Of these, 6 koalas (85.7%) required two hospital admissions for treatment (75% of females (n = 3) and 100% of males (n = 3)), and 1 female koala required three hospital admissions for treatment (25% of females). Of the koalas requiring two hospital admissions for treatment, 100% (n = 6) were due to new infections, and all were released back into the wild. The female koala requiring three consecutive hospital admissions for treatment was determined to have newly acquired infections on each occasion and was euthanized during her final admission due to the development of an iatrogenic condition, CDTS. There was no significant association between the necessity for additional hospital admissions for treatment and the length of chloramphenicol treatment (14 to 27-day versus 28-day course) (*p* = 0.7088), or between the number of admissions with negative short-term outcomes and the length of chloramphenicol treatment (Table 2).

**Table 2:** Outcomes for varying treatment lengths of daily subcutaneous chloramphenicol injections at Endeavour Veterinary Ecology during the koala management program.

Chloramphenicol treatment length (days)	Number of repeat hospital admissions	Number of admissions with negative short-term outcomes
Fourteen	1/7	0/7
Fifteen to twenty-seven	1/15	1/15
Twenty-eight	6/44	2/44

### Ovariohysterectomy in combination with chloramphenicol

A total of 13 female koalas with bilateral reproductive disease (resulting in sterility) received the standard regimen of chloramphenicol treatment at EVE and surgical ovariohysterectomy. Of the 12 koalas released back into the wild after treatment, none developed subsequent chlamydial disease or *Chlamydia* antigen test positivity for the remainder of the koala management program or until their death/cessation of monitoring (median 466 days, range 37–793 days), and all were qPCR-negative at their subsequent and/or final clinical examinations (S1 Table). One koala was euthanized due to procedure-related complications, after developing neurological signs on recovery from anaesthesia, and a partial intestinal volvulus and caecal stasis a few days later.

## Discussion

The MBR koala management program provided valuable data on the short and long-term clinical outcomes for a large number of free-living koalas treated for *Chlamydia* infection and disease. Population viability analyses of this koala population and the population-scale benefits of chlamydial management are reported elsewhere [7, 10]. Those reports indicate that chlamydial disease prevalence was approximately 27% at the commencement of management, and close to 0% in the final year. We report here, the efficacy and benefits of chlamydial disease treatment at the individual koala level. We found the standard treatment regimen of a 14 to 28-day course of daily subcutaneous chloramphenicol injections was highly effective at achieving a microbiological cure and sufficient resolution of clinical signs to result in long-term disease resolution in over 94% of koalas admitted for treatment. These outcomes demonstrate the conservation and animal welfare benefits of intensive population-scale veterinary management to declining koala populations.

Treatment of chlamydial infection and disease during the koala management program was successful in most cases. This contrasts with other studies reporting equivocal clinical outcomes and negative sequelae with chloramphenicol use [21, 23, 24]. Overall, 86.3% of admitted koalas had positive long-term clinical outcomes and were released back into the wild after treatment. When the data were censored to remove non-standard treatments (e.g. short treatment length, treatment with non-chloramphenicol antimicrobials, topical antimicrobial or surgical treatment only), and those that took place at other facilities, the success of treatment rose to 94.8%. These results demonstrate the value of this treatment regimen for successful clinical outcomes for koalas.

The success of the treatment regimen employed during this study can largely be attributed to chloramphenicol use. However, early detection and treatment of iatrogenic conditions and specialised nursing care were also important for positive long-term clinical outcomes, supporting the findings of Griffith and Higgins [24]. Although the chloramphenicol regimen used in this program was not associated with a significant risk of bone marrow suppression or development of CDTS, oxalate nephrosis and candidiasis were detected relatively commonly (though only resulted in mortality in a minority of cases–Fig 1), in contrast to the findings of Govendir *et al*. [22]. These conditions are most likely triggered by compositional changes in individuals’ gastrointestinal microbiome, in particular, suppression or loss of oxalate-degrading bacteria in cases developing oxalate nephrosis [23].

Whilst candidiasis was generally easy to treat with oral anti-fungal therapy, oxalate nephrosis was more challenging. Oral transfaunation using fresh caecal contents, a therapy adopted by us in the latter part of the program, along with supportive fluid therapy resulted in success in a small number of cases and has now been adopted as standard by the authors. No deaths from oxalate nephrosis occurred after the implementation of this treatment regimen. Finally, the prompt euthanasia of severe chlamydial disease-affected koalas with a poor long-term prognosis is an important animal welfare management strategy, and clearly improved the treatment success of the remaining diseased cohort admitted for treatment.

Strain of *Chlamydia*, co-infections, site-specific microbiomes, environmental factors, host genetics and immune factors are all likely to affect the pathogenesis and clinical outcomes following chlamydial infection or exposure in koalas. Notably, in this study, sex did not have a statistically significant impact on clinical outcomes. Koalas with severe chlamydial disease, abnormal blood and bone marrow parameters, and poor body condition have been suggested to be more likely to have co-infections with an immunosuppressive pathogen, namely KoRV sub-type B (KoRV-B) [4, 27]. Further analysis of the specific KoRV infection patterns in these koalas is beyond the scope of our study but may assist in further defining the role of KoRV in the pathogenesis of chlamydial disease and negative clinical outcomes.

Previous studies have indicated that shorter chloramphenicol regimens may be effective against *Chlamydia* in koalas. Markey *et al*. [21] and Govendir *et al*. [22] reported a rapid reduction and cessation of shedding from both the ocular and urogenital tract sites after two weeks of treatment, but 45-day treatment regimens were used in these studies due to common protocols at the time. However, our shorter standard treatment regimen appears to be just as efficacious. Shorter treatments may also improve clinical outcomes for admitted koalas by minimising the risk iatrogenic conditions such as candidiasis, oxalate nephrosis and CDTS, and reduce the costs associated with treatment. Our results show that there was no significant difference in the number of cases requiring a second admission for treatment of *Chlamydia* between koalas receiving a 14 to 27-day versus a 28-day course of chloramphenicol, suggesting a shorter duration of treatment may be sufficient, particularly for cases of mild to moderate ocular disease.

The treatment success achieved in this study not only demonstrates that chloramphenicol administered according to this regimen is safe and efficacious, it provides further indirect evidence that chloramphenicol treatment is able to achieve a permanent microbiological cure. Data derived from ovariohysterectomized koalas suggest that the rate of recrudescence of infection following appropriate chloramphenicol treatment is low (no koalas developed new disease or infections). This supports the contention that koalas requiring multiple admissions for chlamydial treatment were subject to new infections, rather than recrudescence of previous infections, and that infection most commonly occurs via sexual transmission. In contrast, koalas receiving non-standard (non-chloramphenicol) treatments often required further treatment due to treatment failure. However, the role of persistent forms of *Chlamydia* cannot entirely be ruled out [28], or self-limiting infections in cases of ocular disease [29].

Despite the positive clinical outcomes achieved with chloramphenicol treatment, alternatives are warranted due to the decreasing use and availability of chloramphenicol preparations, and the human health risks associated with exposure of sensitive individuals. Doxycycline is being investigated for efficacy and safety in the treatment of chlamydial infection and disease when administered as weekly subcutaneous injections for four weeks. A serine protease inhibitor targeting high temperature requirement A (HtrA) significantly reduced infectious progeny of *Chlamydia* in both *in vitro* and *ex vivo* cell cultures from koalas with disease, and in some cases, was lethal for isolates of both *C*. *pecorum* and *C*. *pneumoniae* [30]. Although Lawrence *et al*. [30] reported low overall toxicity in *ex vivo* tissue cultures, additional research is required to ensure safety *in vivo*, and optimise the formulation. The recombinant Major Outer Membrane Proteins (MOMP) koala *Chlamydia* vaccine shows promise in reducing disease impacts at the population level, prolonging the duration of benefit derived from population-scale veterinary management, and may offer therapeutic as well as preventative benefits [13, 31, 32]. Finally, a greater understanding of chlamydial pathogenesis and epidemiology is an important research priority, including the role of KoRV.

## Supporting information

S1 Table Real-time PCR results for koalas treated during the koala management program. (PDF)

S1 File Clinical data for koalas treated during the koala management program. (XLSX)
