# Supplementary material for: Optimising the short and long-term clinical outcomes for koalas (Phascolarctos cinereus) during treatment for chlamydial infection and disease
Source: PLoS One. 2018 Dec 27;13(12):e0209679. doi: 10.1371/journal.pone.0209679 (PMC6307739; doi:10.1371/journal.pone.0209679)
Supplement: S1 Table — (PDF) [file pone.0209679.s001.pdf]

NAME	SEX	DATE	EXAM TYPE	UGT qPCR (copies/µL)	DATE	EXAM TYPE	UGT qPCR (copies/µL)	DATE	EXAM TYPE	UGT qPCR (copies/µL)	DATE	EXAM TYPE	UGT qPCR (copies/µL)	TREATMENT LENGTH (DAYS)
Andrew	Male	1/08/2014			18/12/2014	Subsequent exam after treatment for cystitis								
Bubbles	Female	9/01/2015	Pre treatment e:	976	10/02/2015	Post treatment exa	BDL	22/04/2015	Subsequent exam	BDL				28
Cailan	Male	4/04/2016	Pre treatment e:	218.4	17/04/2016	Post treatment exa	BDL	8/09/2016	Subsequent exam	BDL				14
Chris	Female	14/11/2014	Pre treatment e:	N/A	15/12/2014	Post treatment exam after OHE a		20/07/2015	Subsequent exam	BDL	18/03/2016	Final vet exam	BDL	28
Circe	Female	22/10/2014	Pre treatment e:	BDL	18/11/2014	Post treatment exam after OHE a		18/01/2017	Final vet exam	BDL				28
CJ	Female	30/10/2014	Pre treatment e:	N/A	5/12/2014	Post treatment exa	BDL	11/01/2017	Final vet exam	BDL				18
Copper	Male	30/12/2014	Pre treatment exam for cystitis											
Daisy	Female	6/01/2014	Pre treatment exam for cystitis											
Damian	Male	7/08/2014			21/08/2014	Post treatment exam for cystitis								
Grumps	Male	1/09/2014	Pre treatment exam for chronic		1/10/2014	Post treatment exam								
Hestia	Female	2/12/2014	Pre treatment exam for reprodu		18/12/2014	Post treatment exa	BDL	10/06/2015	Subsequent and fir	BDL				16
Jeremy	Male	21/10/2014			25/11/2014	Post treatment exam for cystitis								
Kapok	Female	8/01/2014	Pre treatment exam with cystiti		25/06/2014	Subsequent exam after treated for cystitis (before reproductive disease)								
Kapok	Female	5/02/2015	Pre treatment w	135.6	4/03/2015	Post treatment exa	BDL	24/06/2015	Subsequent exam	BDL				19
Karen	Female	22/01/2015	Pre treatment e:	2344	19/02/2015	Post treatment exa	BDL	19/05/2015	Subsequent exam	BDL				28
Kas	Female	26/08/2014	Pre treatment e:	N/A	9/09/2014	Post treatment exa	BDL	25/11/2014	Subsequent exam		8/04/2016	Final vet exam	BDL	14
Kelly	Female	4/02/2016				Euthanased before release								
Marcela	Female	16/09/2014	Pre treatment e:	259.2	13/10/2014	Post treatment exa	BDL	23/02/2015	Subsequent exam	BDL	11/02/2016	Final vet exam	BDL	28
Mary Po	Female	18/09/2014	Pre treatment e:	27280	16/10/2014	Post treatment exa	BDL							28
Shorty	Female	10/10/2014	Pre treatment e:	346.4	28/11/2014	Post treatment exam after OHE a		23/02/2016	Final vet exam	BDL				24
Sophie	Female	14/10/2014	Pre treatment e:	932	12/02/2015	Subsequent exam	BDL	25/02/2016	Final vet exam	BDL				28
Tait	Male	9/09/2014	Pre treatment exam for cystitis											
Tania	Female	17/03/2016	Pre treatment fo	19920	13/04/2016	Post treatment for	BDL	17/01/2017	Final and subsequ	BDL				28
Venom	Female	13/10/2014	Pre treatment e:	77.6	17/11/2014	Post treatment exam after chlora		6/02/2016	Final vet exam	BDL				28
Walt	Male	18/09/2014	Pre treatment e:	280.8	15/10/2014	Post treatment exam		6/05/2015	Subsequent exam	BDL				28

- (pink) OHE koalas who also received a minimum of 14 days of chloramphenicol treatment at EVE
- (yellow) Analysed by other researchers using 16S target
- (grey) Excluded from analysis
